# Denture-Soaking Solution Containing *Piper betle* Extract-Loaded Polymeric Micelles; Inhibition of *Candida albicans*, Clinical Study, and Effects on Denture Base Resin

**DOI:** 10.3390/antibiotics10040440

**Published:** 2021-04-15

**Authors:** Siriporn Okonogi, Pimpak Phumat, Sakornrat Khongkhunthian, Kullapop Suttiat, Pisaisit Chaijareenont

**Affiliations:** 1 Department of Pharmaceutical Sciences, Faculty of Pharmacy, Chiang Mai University, Chiang Mai 50200, Thailand; 2 Research Center of Pharmaceutical Nanotechnology, Chiang Mai University, Chiang Mai 50200, Thailand; sakornratana.k@cmu.ac.th (S.K.); pisaisit.c@cmu.ac.th (P.C.); 3 Interdisciplinary Program in Nanoscience and Nanotechnology, Faculty of Science, Chiang Mai University, Chiang Mai 50200, Thailand; pimpak_p@cmu.ac.th; 4 Department of Restorative Dentistry and Periodontology, Faculty of Dentistry, Chiang Mai University, Chiang Mai 50200, Thailand; 5 Department of Prosthodontics, Faculty of Dentistry, Chiang Mai University, Chiang Mai 50200, Thailand; kullapop.s@cmu.ac.th

**Keywords:** *Piper betle*, polymeric micelles, denture-soaking solution, poloxamer, solubility, anti-candidiasis, PMMA

## Abstract

*Candida albicans* is a common overgrowth in people wearing dentures. Long-term use of antifungal chemicals carries a risk of toxic side effects. This study focused on the edible *Piper betle* extract because of its safety. The broth dilution method was applied for antifungal determination of the ethyl acetate fractionated extract (fEA) and fEA-loaded polymeric micelles (PMF). The PMF was prepared by thin-film hydration using poloxamer 407 as a polymer base. The results found that the weight ratio of fEA to polymer is the main factor to obtain PMF system as a clear solution, nanoparticle sizes, narrow size distribution, negative zeta potential, and high entrapment efficiency. The activity of PMF against *C. albicans* is significantly higher than fEA alone, with a minimum fungicidal concentration of 1.5 mg/mL. PMF from 1:3 ratio of fEA to polymer is used to develop a denture-soaking solution contained 1.5 mg fEA/mL (PMFS). A clinical study on dentures of 15 volunteers demonstrated an 86.1 ± 9.2% reduction of *C. albicans* after soaking the dentures in PMFS daily for 14 days. Interestingly, PMFS did not change the hardness and roughness of the denture base resins. The developed PMFS may serve as a potential natural denture-soaking solution against candidiasis in denture wearers.

## 1. Introduction

*Candida albicans* is a main pathogenic microorganism that causes oral candidiasis. It is commonly found in people who wear dentures. Poor denture hygiene is a potential reason for pathogenic accumulation at the denture devices [1], leading to inflammation at the mucous membrane covered by the denture called denture stomatitis. The symptoms of denture stomatitis are different in each person, including inflammation, burning sensation, bleeding, halitosis, or even no symptoms occur in some cases [2]. The virulence of this infectious disease can lead to mortality by the pathogens that reach the blood circulation [3]. In addition, the severity of oral candidiasis or stomatitis can occur from a decrease in oxygen and saliva flow to the oral tissue covered by dentures. These conditions lead to the production of highly local acidic and anaerobic environment that facilitates *C. albicans* and the overgrowth of other bacteria such as *Streptococcus sanguis, Streptococcus oralis*, and Actinomycetes species [4]. The previous reports on epidemiological studies among denture wearers have shown that the prevalence of denture stomatitis is ranged from 15% to over 70% and is higher among elderly denture users and among women [5] and it will increase in the patients who always wear the denture with poor oral hygiene [1,6].

Polymethylmethacrylate (PMMA) is the material of choice for the denture base. It has many advantages such as low toxicity, good mechanical properties, ease of manipulation, adjustable color, and shade to gum and oral tissue. Moreover, it is also easy to shape and contour for being adaptable with the changing oral structures [7]. However, the porous architectures and the surface roughness that are the hereditary properties of the polymerized PMMA are the main drawbacks of this material such as microorganism accumulation and colonization can easily occur on the denture surface made of this material. Wearing of dentures with a rough and irregular surface has been categorized as one of the major factors related to the increasing of tissue inflammation in denture wearers called denture stomatitis [8]. Therefore, well-polished dentures from brushing and daily soaking in the appropriate solutions have been recommended to the patients for eradicating the accumulated pathogenic biofilms and removing the newly accumulated pathogens on the denture surface [9].

Nystatin, amphotericin B, and imidazole drugs such as fluconazole and miconazole are the medicines that are used for the treatment of fungal infection in the oral cavity [10]. Besides, certain chemicals such as chlorhexidine (CHX) or hypochlorite are reported to have the ability against pathogenic fungi. Denture-soaking solutions containing these antifungal agents are attracted as an alternative or complementary protocol, particularly for some patients who have limitations for mechanical cleansing procedures especially the group of geriatric or disabled patients [9]. However, these chemicals present many side effects after long-term applications [11] such as changing oral taste, increasing calculus formation [12], and affect the denture by causing erosion of the metal part of the dentures [13]. Therefore, plant extracts, particularly from edible parts with antimicrobial activity receive increasing interest as they are natural and fewer side effects.

**Figure 1:**
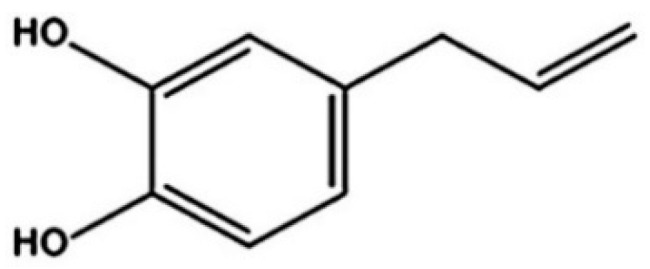
Chemical structure of APC.

Previous studies reported the potential of medicinal plants against oral pathogens e.g., *Caesalpinia sappan* showed antibacterial activity against *Streptococcus mutans* [14], *Glycyrrhiza uralensis* showed antibacterial activity against *Porphyromonas gingivalis* [15] and *Citrus limonum* showed antibacterial activity against *Enterococcus faecalis* [16]. Our previous study found that ethyl acetate fractionated extract (fEA) of *Piper betle*, one of the potential medicinal plants in the family Piperaceae possessed strong inhibitory activity against *C. albicans* [17]. Moreover, an active phenolic compound, known as 4-allylpyrocatechol (APC), with a chemical structure as shown in Figure 1, was isolated from fEA. APC also showed strong inhibition against several oral pathogens. However, both APC and fEA as well as most plant active compounds are still less developed into modern pharmaceutical products and less applicable in clinical uses because of their poor properties such as low aqueous solubility, rapid metabolism, and instability [18].

Many techniques can be used to increase the solubility of poorly water-soluble active ingredients including the physical and chemical modifications of the compounds or other methods such as particle size reduction, crystal engineering, complexation, solid dispersion, co-solvency, micellization, salt formation, cryogenic techniques, and so on [19]. Polymeric micellization is an interesting technique based on micellization. By using suitable polymers and preparing conditions, nanoparticles of polymeric micelles (PMs) can be obtained. PMs can solve the solubility problems of hydrophobic drugs and act as potential drug delivery vehicles that can increase drug concentration in blood circulation and provide the drugs to reach the targeting sites [20]. According to mucoadhesion property of some polymers used in PMs, this system is considered to be the potential carriers for the local delivery of drugs in mucosal surfaces e.g., pulmonary, eyes, and gastrointestinal tract [21]. In addition, PMs have also been reported on its potential to penetrate through fungal membrane and releasing the entrapped drug inside the fungal cells [22]. PMs can be self-assembled, formed in an aqueous solution above a critical micelles concentration (CMC) of the polymer. The hydrophobic drug will be incorporated into the hydrophobic core of the micelles that lead to increase solubility whereas the hydrophilic shell stabilizes the micelles against aggregation [23,24]. Poloxamer is a non-ionic triblock copolymer and composed of polyethylene oxide (PEO) as a hydrophilic part and polypropylene oxide (PPO) as a hydrophobic part. According to this amphiphilic structure, PMs of poloxamer can be formed. Poloxamer is approved by the U.S. FDA as one of the suitable biomaterials for drug delivery [25]. Previous studies reported the potential of poloxamer on enhancing the solubility and activity of certain active compounds or crude extracts [26,27].

Even when both APC and fEA possess strong antimicrobial activity, fEA was selected to use as an active compartment in the present study because the yield of fEA was significantly higher than APC (data not shown). Moreover, it was reported that many plant extracts showed higher advantages on biological activities and stability than their individual pure compound due to the synergistic effects of the components in the extracts [28]. The development of fEA-loaded poloxamer PMs (PMF) is explored in the present study in order to enhance the aqueous solubility as well as antifungal activity of fEA. The denture-soaking solution containing PMF (PMFS) was further developed. For the clinical study, the efficiency of this solution on antifungal activity was investigated using dentures of 15 volunteers. The effects of PMFS on surface roughness and hardness of PMMA polymer as a denture base resin were also evaluated.

## 2. Results and Discussion

### 2.1. Development of PMF

**Figure 2:**
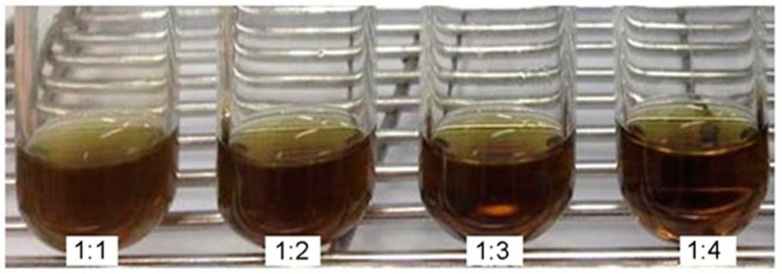
Outer appearance of PMF formulations in various ratios of fEA to P407 polymer.

The amphiphilic block polymers containing hydrophilic and hydrophobic chains can self-assemble into PMs at or above their CMC [29]. CMC of poloxamer 407 (P407) used in the present study is approximately 6.9 × 10^−5^ M (0.8694 mg/mL) [30]. The HPLC analysis confirmed that APC was the major composition of the extracted fEA (data not shown) and the amount of APC in the extract was approximately 26%, similar quantity as previously reported [17,31]. After adding water to the fEA-polymer films, fEA could be entrapped into the forming PMs during the self-assembly process, and the PMF nanoparticles were obtained. The PMF formulations obtained from several weight ratios of fEA to P407 presented as a dark-brown solution as shown in Figure 2. As P407 solution is colorless, the color of the PMF formulations was due to the dissolved fEA. It was visually observed that the PMF systems of 1:3 and 1:4 ratios were completely clear, suggesting that the amount of P407 was sufficient to completely entrap all fEA. However, turbidity could be obviously seen in the systems of 1:1 and 1:2 ratios after freshly prepared and precipitation occurred after 24 h standing at room temperature.

When turbidity occurs, it can cause a light obstruction which includes the scattering and absorption of light by solute molecules or particles in the systems [32]. Measuring light transmittance can indirectly indicate the turbidity of the systems. In the present study, using the transmittance mode of the spectrophotometer, the turbidity of the systems can be calculated as in Equation (1):

Turbidity (%) = 100 − %T,(1)

where T is the transmittance of the system. The results are shown in Table 1. It was found that the ratio of fEA to P407 significantly affected the turbidity of the PMF system. The system of 1:1 ratio showed the most turbidity, followed by that of 1:2 ratio, whereas the systems of 1:3 and 1:4 ratios were the same and far less than that of 1:1 and 1:2 ratios. These results confirmed that observed visually. These results suggested that the quantities of P407 in the PMF systems of 1:1 and 1:2 ratios were inadequate. The results also showed that the interference from the color of fEA. The clear solution of fEA in ethanol showed the transmittance to be only 9.28 ± 0.01, likely it had 90.72 ± 0.01% turbidity, for which indeed the system is visually very clear. This result indicated that the color of samples, resulting from dissolved compounds, can affect a turbidity measurement. Our results were in accordance with the previous report [33].

The results of particle analysis of PMF using photon correlation spectrophotometer (PCS) as shown in Table 2 demonstrates that the particle size of PMF obtained from the 1:3 and 1:4 ratios was larger than those obtained from the 1:1 and 1:2 ratios. Excessive polymer could be located at the outer part of the nanoparticles by some forces [34]. The larger size of the PMF in either 1:3 or 1:4 system confirmed the sufficient polymers and was considered that the excess P407 could form hydrogen bonding among the hydrophilic PEO groups on the particle surface and hydrophobic forces of the hydrophobic PPO group among the polymer chains. It was also noted that the PMF system of 1:3 ratio possessed a smaller particle size and narrower sized distribution than that of 1:4 ratio. The narrow size distribution with a polydispersity index (PdI) of 0.20 ± 0.01 of the 1:3 ratio system indicated good homogeneity of the distributed particles [35].

Zeta potential is an electrokinetic potential of the particles that are distributed in the aqueous medium. This potential is existing at the hydrodynamic plane of shear between the Stern plane and the end of the diffuse layer and associated with a realistic magnitude of surface charge of the particles. The zeta potential plays an important role in the properties of nanoparticles such as particle size, targeted drug delivery, and stability [36]. The positive or negative zeta potential value affects a repulsive force resulting in prevent aggregation of the particles in colloid or suspension systems [37,38]. The results in the present study showed that the zeta potential of PMF obtained from all ratios was a negative charge with values ranging from ‒3.54 ± 0.56 to ‒5.18 ± 0.08 mV. The low value of zeta potential might be influenced by the nature of the non-ionic polymer of P407 [39]. Comparing between the PMF systems of 1:3 and 1:4 ratios which all drug loaded could be entirely entrapped, the 1:3 ratio was more suitable since it yielded significantly smaller particle size and a narrower size distribution, with slightly higher zeta potential value. This system was selected for further investigations.

### 2.2. In Vitro Antifungal Activity

#### Minimum Inhibitory Concentration (MIC) and Minimum Fungicidal Concentration (MFC)

The in vitro antifungal activity against two *C. albicans* strains of the selected PMF was evaluated in comparison with the unentrapped fEA using a broth dilution method. The results expressed as MIC and MFC values are shown in Table 3. A lower value of MIC and MFC represents a higher fungal inhibitory activity and fungicidal potential, respectively. It was found that the MIC values of PMF against both fungal strains were the same as that of fEA suggesting that their inhibitory activities against these fungi were the same level. However, the MFC values of PMF were significantly lower than that of fEA, indicating that the fungal killing effects of PMF was significantly higher than that of fEA. It was previously reported on the beneficial of PMs on cellular uptake into the pathogenic cells and enhancement of permeability through biological membranes of fungal cells which promotes the antifungal activity [40,41]. The result in the present study confirmed the potential of PMF on enhancing cellular uptake of the test *C. albicans* strains. Antimicrobial activity is usually regarded as fungicidal if the MFC/MIC ratio is <4 and fungistatic if the ratio is >4 [42]. A substance that can stimulate cell death is categorized as a fungicidal agent [43] whereas a fungistatic agent is supposed only to inhibit cell division [44]. Moreover, fungicidal agents usually demonstrate more rapid clearance of pathogens and reach to the effective therapeutics when compared to fungistatic agents [45]. Therefore, agents possess fungicidal activity are preferable to those possess fungistatic effect. The results of the present study obviously indicated that the PMF and fEA could act as a fungicidal agent against *C. albicans.* The results showed that nystatin has the possibility to be either a fungicidal or fungistatic agent. It has been reported that the antifungal activity of nystatin is depending on the concentration of the drug. At low concentrations, nystatin shows a fungistatic effect to prevent growth of a wide variety of fungi, but at higher concentrations, its activity becomes fungicidal effect [46]. From PMF at the concentration of 1.5 mg fEA/mL showed efficacy against both strains of *C. albicans*, the empty PMs (PMs without fEA) which prepared from P407 in a same amount that used to prepared the efficient concentration of PMF (1.5 mg fEA: 4.5 mg P407) showed no efficacy to inhibit and kill strains of *C. albicans*.

### 2.3. Visualization of Morphological Alterations

**Figure 3:**
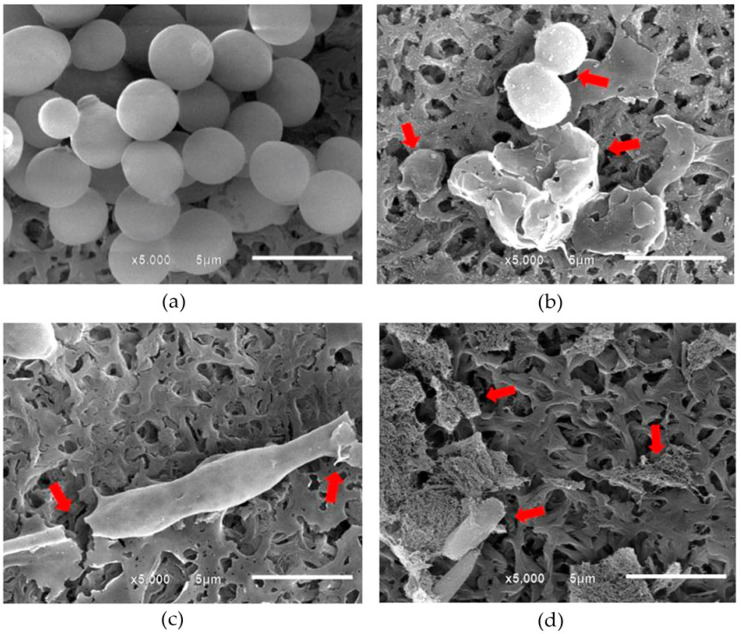
SEM micrographs of *C. albicans*; (**a**) untreated cells; (**b**) treated with PMF; (**c**) treated with fEA; (**d**) treated with nystatin. The red arrows indicate the destruction of *C. albicans* cells.

Generally, the change from a spherical shape of yeast form to budding or tubing formation prior to the hyphal form of *C. albicans* was considered to be a key point in pathogenesis of this pathogen. In the present study, the morphological alterations of the tested *C. albicans* after contacting with the selected PMF at the concentration of 1.5 mg fEA/mL were investigated, in comparison with the untreated cells and those contacting with Nystatin (0.1 mg/mL), using scanning electron microscope (SEM). The SEM micrographs of the untreated *C. albicans* as shown in Figure 3a demonstrated a regular shape of the mature yeast cells and smooth surface as well as the normal budding formation indicating the normal extend outgrowth from the mother cells. However, after contact with PMF, cellular swelling and damage was observed (Figure 3b). Moreover, the cell population was significantly reduced in comparison with the untreated cells. The fEA could also demolish the cells. A long-developed tube shape of *C. albicans* cells was found, likely an abnormal and incomplete budding formation occurred, and some were broken (Figure 3c). The SEM image of *C. albicans* after contacting with nystatin is shown in Figure 3d. It was found that the cells were broken into pieces. It has been reported that nystatin could damage *Candida* cells by binding with ergosterol, a major component of the fungal cell membrane and lead to cell death [47]. The results in the current study clearly showed that the morphological cell change of *C. albicans* caused by PMF, fEA, and nystatin was different. Therefore, the mechanism of damaging *C. albicans* cells of PMF and fEA might be different from nystatin. It has been reported that the phenolic compounds from plants can cause microbial cell death by generating superoxide which produced by a single electron reduction of molecular oxygen and damage microbial DNA. From the chemical structure, APC which is a major active compound in fEA is characterized as a phenolic compound [48]. Therefore, the probable mechanism of PMF and fEA against *C. albicans* might be due to the generation of free radical species to damage DNA of the cells.

### 2.4. Preparation and Characterization of PMFS

**Figure 4:**
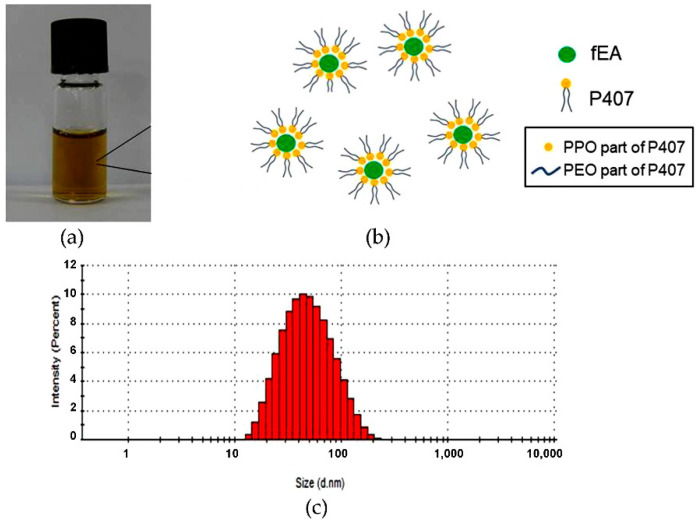
Characterization of PMFS; (**a**) physical appearance; (**b**) the self-assembly formation of fEA-loaded PMs; (**c**) histogram of particle size and size distribution after determined using photon correlation spectroscopy (PCS).

The developed aqueous denture-soaking solution, a so-called PMFS, containing PMF with 1.5 mg/mL fEA appeared as a clear solution with a light brown color as shown in Figure 4a. It is noted that fEA was water insoluble; however, after entrapping into the PMs, the obtained PMF could dissolve completely in aqueous systems. The developed PMFS should contain fEA at least 1.5 mg/mL because it was an MFC value of this substance against *C. albicans* as the result demonstrated above. In the aqueous solution, P407 could self-assemble into nanoscale micelles [49]. The hydrophobic fEA could be incorporated into these micelles core to create PMF particles as shown in Figure 4b. Particle analysis using PCS demonstrated that the particle size of PMF in PMFS was 49.80 ± 0.42 nm and narrow size distribution with a PdI value of 0.20 ± 0.01. The histograms of size distribution by the intensity of the selected PMF in PMFS are shown in Figure 4c. These results confirmed that the PMF particles in the obtained PMFS system was in nano-scale range with a homogenous distribution. Besides, the PMFS showed the zeta potential of –5.39 ± 0.57 mV, supporting the zeta potential of PMF. Entrapment efficiency (EE) was determined to evaluate the efficiency of PMF on entrapment of fEA. The determination of EE can be performed by either direct or indirect method. In the direct method, the nanoparticles are destabilized in a suitable solvent and measured the amount of the entrapped drug in the particles [50]. The indirect method is the procedure that determines the unentrapped drug in the supernatant or dispersion medium, then the entrapped drug can be calculated using Equation (2):

EE (%) = (D_o_ − D_u_)/D_o_ × 100, (2)

where D_o_ is the amount of drug used for loading and D_u_ is the amount of the unentrapped drug in the supernatant. The indirect method suffers from many limitations e.g., a low concentration of drug in the supernatant may impose some limitations in the detection process and there might be certain interfering substance in the supernatant [50,51]. Thus, the direct method was applied to determine EE of PMF in the current study. After destabilizing the PMF particles by absolute ethanol, the UV absorption of fEA was recorded and the quantity of the entrapped fEA was calculated using a linear Equation (3) with an *r*^2^ = 0.9999;

y = 0.01224x + 0.28347,(3)

where y is the absorption of fEA and x is the concentration of fEA (µg/mL). The results showed that the PMF particles obtained from the selected 1:3 ratio could completely entrapped fEA with the EE of 100.13 ± 1.54%.

### 2.5. Clinical Study

**Figure 5:**
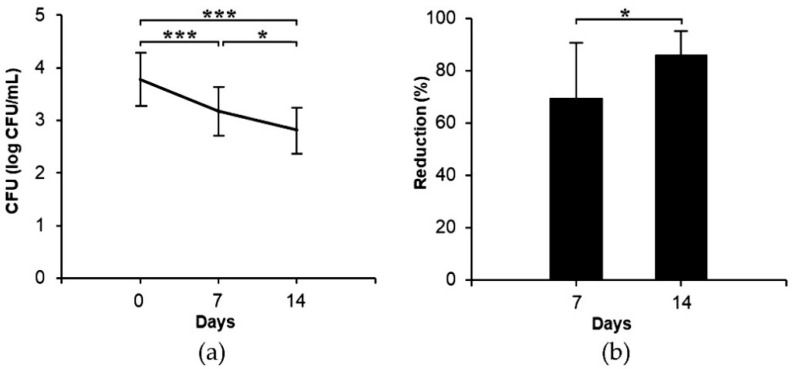
Efficacy of PMFS against *C. albicans* in clinical study after 7-day and 14-day period of applications; (**a**) colonies of *C. albicans* in each day; (**b**) % cell reduction of *C. albicans* in Day 7 and Day 14. Error bars represent the mean ± SD (n = 15). * *p* < 0.05 and *** *p* < 0.001; paired t-test.

After soaking the regularly used dentures in the developed PMFS continuously 8 h at nighttime every day for 14 days, the dentures were investigated for *C. albicans* growth. The results presented as colony forming units per milliliter (CFU/mL) are shown in Figure 5. It was found that the developed PMFS could significantly reduce the growth of *C. albicans* and the fungal reduction was directly related to the time of the denture in contact with PMFS. For example, *C. albicans* colonies at Day 14 (2.81 ± 0.45 log CFU/mL) and at Day 7 (3.17 ± 0.46 log CFU/mL) were significantly less than at Day 0 (*p* < 0.001) as shown in Figure 5a. The antimicrobial potential of PMFS against *C. albicans* could be expressed as a percentage of cell reduction which could be calculated from Equation (4):

Cell reduction (%) = (C_i_ ‒ C_t_)/C_i_ × 100, (4)

where C_i_ is the number of *C. albicans* colonies (CFU/mL) at Day 0 and C_t_ is the number of *C. albicans* colonies (CFU/mL) at Day 7 or Day 14. The results as shown in Figure 5b clearly demonstrated that cell reduction according to PMFS at Day 14 (86.1 ± 9.2%) was significantly (*p* < 0.05) higher than that at Day 7 (69.6 ± 20.9%). The possible mechanism that promotes the activity of PMFS to inhibit *C. albicans* on the dentures may be due to the marvelous property of the micelles which present a superior penetration ability to pass through the polymeric matrix of dentures and interact with the membrane of *C. albicans* [41]. Polishing with toothbrush has been recommended after soaking the dentures with an agent to enhance the reduction of pathogens. It was previously reported that soaking the dentures in coconut soap, sodium perborate, or 2% CHX, followed by toothbrush polishing, the population of *C. albicans* could be reduced by time-dependent manner. Cell reductions of these three systems calculated at Day 7 were 32%, 46%, and 69%, respectively [52]. In comparison with the current study, the developed PMFS showed significantly high potential on reduction of *C. albicans* as it gave higher cell reduction than those that were previously reported and even without polishing. Therefore, PMFS is a promising denture-soaking solution that can reduce *C. albicans* and decrease stomatitis.

### 2.6. Effects of the PMFS on Mechanical Properties of Acrylic Resin

The physical and mechanical properties of denture base material, particularly the hardness and the roughness, are classified as crucial parameters that affect the oral tissue of the denture wearer. The changes in hardness and roughness of the acrylic denture base may initiate oral tissue irritation or inflammation. The denture with a roughing surface usually relates to the increasing of microorganism accumulation especially *C. albicans* that is the most common and main pathogen of candidiasis [53]. The previous study reported that the surface roughness of denture base material which is greater than 0.2 µm can promote the formation of biofilm and result in a high concentration of microorganism colonization on the denture surface [54,55]. Furthermore, the alteration of surface hardness of denture base material may lead to detrimental changes related to the durability of the denture in oral environment. From the above reasons, the effect of denture-soaking solution on the hardness and roughness of acrylic denture base material must be a concern as the important parameters and must be realized in the development of denture-soaking solution. The inhibitory effects of various agents, e.g., sodium hypochlorite (NaOCl), glutaraldehyde, and sodium perborate against *C. albicans* on acrylic resin specimens were previously compared. It was reported that sodium perborate possessed good inhibitory activity but gave the highest effect on acrylic resin by increasing its surface roughness [56]. In the current study, the Vicker’s hardness value and the surface roughness value of PMMA specimens before and after immerging in PMFS, 0.5% NaOCl as a positive control, and distilled water as a negative control for 21 cycles of 8-h soaking are shown in Table 4. It was found that the surface hardness of PMMA after soaking in all groups did not show a statistically significant difference when compared with their initial condition (*p* > 0.05). All groups of soaking solutions did not cause the significantly differences in surface hardness of the PMMA specimens (*p* > 0.05). The surface roughness values (µm) behaved the same as the surface hardness values. They demonstrated the non-statistically significant difference when comparing between before and after immersion in all types of soaking solutions (*p* > 0.05). The surface roughness of PMMA specimens which were immersed in PMFS was not significantly different from the groups that immerged in 0.5% NaOCl and distilled water (*p* > 0.05). The average surface roughness values after completion of soaking in all groups were less than 0.2 µm.

## 3. Materials and Methods

### 3.1. Preparation of fEA

The fresh leaves of *P. betle* were collected from the northern area of Thailand. The voucher specimen of this plant with a reference number of 008612 has been deposited in the Herbarium of the Faculty of Pharmacy, Chiang Mai University, Thailand. The leaves were dried in a hot air oven at 50 °C for 24–48 h. The dried leaves were ground into a fine powder. For extraction, hexane was the first solvent used to macerate the fine powder of *P. betle* leaves. The mixture was filtered through Whatman No. 1 filter paper. Then, the dried macerated residue was further extracted with ethyl acetate. The fEA was obtained after solvent was removed and kept in the refrigerator for further use. In addition, the obtained fEA was subjected to HPLC analysis using the method previously described [17].

### 3.2. Development of PMF

P407 was applied as a polymer base to create the PMs by using thin-film hydration method as previously described [26] with some modification. Briefly, fEA and P407 were separately dissolved in absolute ethanol to prepare the stock solutions of 50 mg/mL extract and 100 mg/mL P407. The stock solutions were mixed to obtain the working solutions of extract and P407 in the weight ratios of 1:1, 1:2, 1:3, and 1:4. The solution was transferred to a round bottom flask and absolute ethanol was evaporated using rotary evaporation at 45 °C for 15–30 min to obtain extract-polymer thin-film. The fEA-loaded PMs was self-assembly formed by dispersed the film in water using a sonicator (WUC-A02H, Daihan Scientific, Gangwon-do, Korea), and then a clear solution of PMF was obtained. Non-entrapped micelles were removed by filtration through 0.22 µm filter.

### 3.3. Particle Analysis

The particles of PMF were analyzed for their size, size distribution, and zeta potential by PCS using a Zetasizer Nano ZS (Malvern, Worcestershire, UK). The PMF formulations were dispersed in water and transferred into an ordinary disposable cuvette, then subjected to the PCS and detected at a fixed angle of 173° at 25 °C. The particle size was expressed as an average diameter of the particles in nm and a size distribution was expressed as a polydispersity index (PdI). Zeta potential of the particles was also determined by PCS at 25 °C. The samples were transferred into DT51070 folded capillary cells (Malvern, Worcestershire, UK) and subjected to the PCS. All measurements were performed in triplicate.

### 3.4. Turbidity Measurement

The turbidity profiles of the PMF formulations of all ratios of fEA to P407 after freshly prepared were determined using a UV/V is spectrophotometer (Model 2450, Shimadzu, Tokyo, Japan) at a wavelength of 630 nm as a method previously described [57] with some modification. The samples were put into a quartz cuvette before subjected to spectrophotometer and determined using a transmittance mode (%T). All measurements were done in triplicate.

### 3.5. Candida Strains and Growth Condition

Two reference strains of *C. albicans* ATCC 10231 and *C. albicans* ATCC 90026 obtained from the Faculty of Dentistry, Chiang Mai University were used in this study. Both strains were cultured in a sabouraud dextrose broth (SDB; Difco, Sparks, MD, USA) at 37 °C for 24 h before testing. The suspension of *C. albicans* was adjusted to 0.5 McFarland turbidity standard using a McFarland densitometer (DEN-1 Biosan, Riga, Latvia), which equals 1 × 10^6^ (CFU)/mL.

### 3.6. In Vitro Antifungal Activity

This experiment was performed using the broth dilution method. The test samples, fEA in DMSO and the selected PMF were prepared in a 96-well plate via a two-fold dilution method using SDB media as a diluent to obtain two-series of fEA concentrations (0.125–2 mg/mL and 0.09–24 mg/mL). Nystatin in the concentration range of 0.0031–20 mg/mL was used as a positive control. The empty PMs prepared from P407 in a same amount that was used to prepare PMF which provide the efficacy against *C. albicans* were also determined. The concentration of each strain of *C. albicans* was adjusted to 1 × 10^4^ CFU/mL before adding to the wells of the 96-well plate. The plate was then incubated at 37 °C for 24 h. After incubation, the lowest concentration of the samples that can inhibit *C. albicans* was recorded as MIC. The dilutions in the wells were streaked on the surface of sabouraud dextrose agar (SDA; Difco, Sparks, MD, USA), and further incubated in the same condition as in the determination of MIC. The lowest concentration of the samples without *C. albicans* on the SDA was recorded as MFC. The experiment was performed in triplicate.

### 3.7. Visualization of Morphological Alteration

The strain of *C. albicans* ATCC 90028 was selected as a model study to investigate the morphological alterations. The possible morphological change of *C. albicans* cells was observed using SEM (JEOL JSM-6610LV, Tokyo, Japan) by the method previously described [58]. Culture suspension at *C. albicans* concentrations of 1 × 10^4^ CFU/mL was added to the PMF formulation and fEA solution (in 3% DMSO) with the same fEA concentration of 3 mg/mL in the 6-well plates to provide the final fEA concentration of 1.5 mg /mL in each. Nystatin at the final concentration of 0.05 mg/mL was used as a positive control. The plates were incubated at 37 °C for 24–8 h. Then, the suspension in each well was filtered through a nylon membrane and the microbial cells were fixed with 2% glutaraldehyde (Merck, Darmstadt, Germany) in phosphate buffer solution pH 7.4 (PBS) containing 137 mM NaCl, 10 mM Na_2_HPO_4_, 2.68 mM KCl and 1.84 mM KH_2_PO_4_. The cells were washed to remove glutaraldehyde and other suspended agent with PBS, and then dehydrated in the increasing ethanol aqueous solutions as follows; 50, 70, 85, 95, and 100%, for 10 min each. The chips of nylon membranes that covered with the *C. albicans* cells after dehydration were dried by critical point drying technique. After that, these chips were mounted on an aluminum stub and coated with gold in a sputter coater (JEOL JFC-1100E, Tokyo, Japan). The cells on the chip were observed at 15 kV accelerating voltage in the SEM with a magnification of 5000×.

### 3.8. Preparation and Characterization of PMFS

As fEA at a concentration of 1.5 mg/mL possessed strong activity against *C. albicans*, this concentration was then applied to develop PMFS. fEA was loaded into P407 micelles with the selected ratio of fEA to P407 to provide a final concentration of 1.5 mg fEA/mL. The obtained PMF particles were investigated for particle size, size distribution, and zeta potential using PCS in the same manner as described in Section 3.3. The EE of PMF in the PMFS was determined using a method as previously described [59] with some modification. The 100 µL of PMFS was dispersed in 900 µL of absolute ethanol and it was then mixed using a vortex mixture for destabilization of PM particles. Then, the solution was centrifuged with 13,000 rpm for 10 min. The absorption of fEA in the supernatant of the solution was determined using UV spectrophotometer (Model 2450, Shimadzu, Tokyo, Japan) at the wavelength of 280 nm and calculated using the standard curve of fEA constructed from the fEA concentration range of 0–200 µg/mL.

### 3.9. Clinical Study

**Figure 6:**
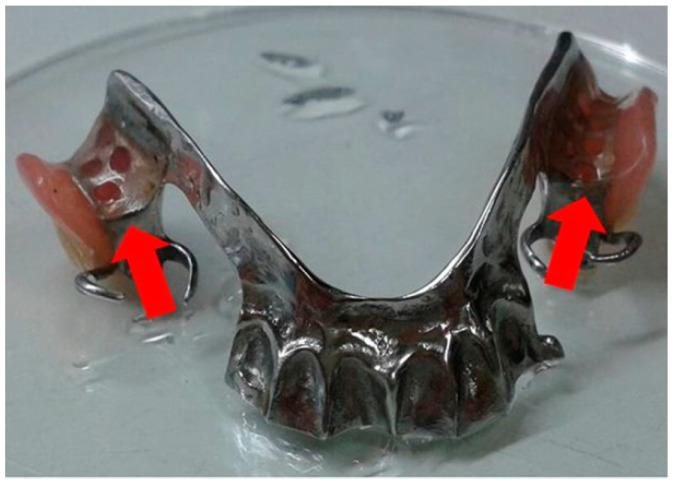
The collected areas from the inner resin base of the dentures.

The clinical study was conducted in accordance with the Declaration of Helsinki, and the protocol was approved by the Human Experimentation Committee, Faculty of Dentistry, Chiang Mai University with a certificate of ethical clearance no. 02/2015. Fifteen healthy adult volunteers, who have worn a denture for at least one year, participated in this study. All volunteers gave their informed consent for inclusion before participating in the study. The potential individuals of participants were excluded if they used the denture cleansing product, smoked, were pregnant or lactating, or had medical conditions, e.g., being treated with antibiotics, anti-inflammatory, or corticosteroid drugs. The application of the PMFS in the clinical study was achieved by soaking of the volunteer’s denture in this solution (approximately 50 mL) overnight (8 h) for 14 days. The analyzed samples were collected from the inner resin base of the dentures as shown in Figure 6 by using a cotton swab and kept in 5 mL normal saline solution (NSS). Then, 20 µL of the collected samples in NSS were spread on SDA and cultured with aerobe condition at 37 °C for 24 h. The colonies of *C. albicans* in the collected samples were determined in the unit of CFU/mL. The samples were collected from the denture’s volunteers three times, before using the PMFS (Day 0), after using the PMFS for 7 days (Day 7) and 14 days (Day 14), respectively.

### 3.10. Effects of PMFS on Physical and Mechanical Properties of Acrylic Resin

The 20 × 20 × 3 mm heat cured PMMA specimens were prepared and embedded into a plastic tube. The prepared specimens were immersed in 37 °C distilled water for 50 h to remove the excessive monomer as the recommendation following the standard procedure of the American Dental Association Specification no.12 [60]. The prepared specimens were assessed using 50× microscope (CK40-F200, Olympus, Tokyo, Japan) and only the specimens without crack and air bubble were selected for further step. The PMMA surface of the selected specimens was polished by waterproof abrasive paper (TOA, Bangkok, Thailand) with progressively increasing grit of 600, 800, 1000, and 1200 in a polishing machine (MoPao 160E, Laizhou Weiyi Experimental Machinery Manufacture Co., Ltd., Shandong, China). The polished specimens were sonicated in distilled water for 5 min to remove the remaining abrasive particles.

According to the soaking solutions PMFS, 0.5% NaOCl and distilled water, were classified as testing, positive and negative control solutions, respectively. The specimens were randomly divided into three groups (20 specimens/group). The 8-h soaking protocol represented a real-time use for denture-soaking during the nighttime was performed on each specimen. After finishing each soaking cycle, the specimen was taken out from the soaking media and rinsed with distilled water for 1 min. The soaking solution was discarded and replaced with the new one before starting a new cycle. The procedure was repeated until reaching the 21 cycles of denture-soaking solution. The hardness and the roughness of PMMA specimens were investigated before and after finishing the immersion procedure.

#### 3.10.1. Surface Hardness Testing

The surface hardness measurements before and after soaking for 21 cycles were performed for all specimens with a microhardness tester (STARTECH SMV-1000 + LCD, Guiyang Sunpoc International Trade Co., Ltd., Guiyang, China). Two diagonals of the indentation left in each specimen (d1 and d2) made by a diamond indenter point in the shape of a square-based pyramid under a 0.49 N load and a 10-sec dwell time was measured using a microscope and their average values (d) were calculated. Three measurements were made at different points and the mean value was recorded. The Vickers hardness (hv) value of each specimen was calculated using Equation (5):

hv = 2Fsin(136°/2)/d^2^, (5)

where F = loading force (kgf), d = arithmetic means of the two diagonals in mm.

#### 3.10.2. Surface Roughness Testing

The roughness of the PMMA surface was measured before and after soaking for 21 cycles by a contact profilometer (Mitutoyo, Aurora, IL, USA). The measurement was determined by moving a 2-μm radius diamond stylus of the profilometer across the specimen surface for 2.4 mm at 0.5 mm/s with a cutoff of 0.8 mm. The roughness measurement was performed in different positions for three times and the mean roughness value was calculated.

### 3.11. Statistical Analysis

All experiments were done in triplicate and the data were expressed as mean ± SD. The data were statistically analyzed using software SPSS version 17.0 for windows with Tukey’s honestly significant difference (HSD) test, a statistical tool used to determine the relationship between two sets of data. Whereas Paired sample T-test was applied for comparing the results of pre- and post–study of a clinical study. The homogeneity of variance and the normality of the hardness and roughness data were tested with the Levene’s and the Lilliefors test, respectively. The statistical significance was evaluated by one-way analysis of variance (ANOVA) with 95% confidence.

## 4. Conclusions

The present study demonstrates a high potential antifungal activity of fEA, an active extract of *P. betle*, against *C. albicans*. From the insight activity, fEA can act as a fungicidal agent against *C. albicans*. The water-soluble limitation of fEA can be solved by entrapment in polymeric micelles of poloxamer 407. The most suitable fEA-loaded polymeric micelle or PMF is composed of 1:3 weight ratio of fEA to poloxamer 407. This PMF system possesses nano-size particles with high entrapment efficiency and suitable for preparing the denture-soaking solution, PMFS. The result from a clinical study shows that this solution possesses high efficiency to reduce *C. albicans* colonies on the denture base after 14 days of applications without any effects on either hardness or roughness of a denture base material. It can be concluded that the obtained PMFS is an effective system of potential *P. betle* extract for use as a denture-soaking solution for prevention and treatment of denture stomatitis caused by *C. albicans*.

## Figures and Tables

**Table 1:** Effect of fEA:P407 ratio on light transmittance and turbidity.

fEA:P407. Ratio and Controls.	Transmittance * (%)	Turbidity * (%)
1:1	27.99 ± 0.02 ^d^	72.01 ± 0.02 ^c^
1:2	29.82 ± 0.01 ^c^	70.18 ± 0.01 ^b^
1:3	35.34 ± 0.01 ^b^	64.66 ± 0.01 ^a^
1:4	35.51 ± 0.01 ^b^	64.49 ± 0.01 ^a^
P407 solution	99.46 ± 0.02 ^a^	0.95 ± 0.02 ^a^
Deionized water	99.45 ± 0.05 ^a^	0.55 ± 0.05 ^a^
fEA in ethanol	9.28 ± 0.01 ^e^	90.72 ± 0.01 ^e^

* Data are presented as mean ± SD of three replicates. Different letters indicate significant difference between treatment groups (*p* < 0.05).

**Table 2:** Particle size, PdI, and zeta potential of PMF formulations.

fEA:P407 (Weight Ratios)	Particles Size * (nm)	PdI *	Zeta Potential * (mV)
1:1	42.26 ± 0.09 ^b^	0.32 ± 0.01 ^c^	−3.54 ± 0.56 ^c^
1:2	41.11 ± 0.28 ^a^	0.27 ± 0.02 ^b,c^	−4.42 ± 0.11 ^a,b^
1:3	45.85 ± 2.27 ^c^	0.20 ± 0.01 ^a^	−5.18 ± 0.08 ^a^
1:4	55.26 ± 1.05 ^d^	0.34 ± 0.02 ^a,b^	−4.67 ± 0.72 ^a,b^

* Data are presented as mean ± SD of three replicates. Different letters indicate significant difference between treatment groups (*p* < 0.05).

**Table 3:** MIC and MFC values (mg/mL).

Samples	*C. albicans* ATCC 10231			*C. albicans* ATCC 90028		
	MIC	MFC	MFC/MIC Ratio	MIC	MFC	MFC/MIC Ratio
PMF	1.0	1.0	1.0	1.0	1.5	1.5
fEA	1.0	2.0	2.0	1.0	2.0	2.0
Nystatin	0.0006	0.0024	4.0	0.0031	0.0124	4.0

**Table 4:** Effect of solutions on mechanical properties of acrylic resin after immersion for 21 cycles.

Solution	Vicker’s Hardness *		Surface Roughness * (µm)	
	Before	After	Before	After
PMFS	19.65 ± 1.19 ^a^	18.03 ± 0.78 ^a^	0.14 ± 0.02 ^a^	0.15 ± 0.03 ^a^
0.5% NaOCl	19.46 ± 0.80 ^a^	19.96 ± 0.84 ^a^	0.14 ± 0.02 ^a^	0.16 ± 0.03 ^a^
Distilled water	19.29 ± 0.73 ^a^	18.52 ± 0.74 ^a^	0.14 ± 0.02 ^a^	0.15 ± 0.03 ^a^

* Data are presented as mean ± SD of three replicates. Lowercase letters indicate significant difference between treatment groups (*p* < 0.05).

## Data Availability

All data are available upon request.

## References

[1] Dar-Odeh N.S., Shehabi A.A. (2003). Oral candidosis in patients with removable dentures. Mycoses.

[2] Gleiznys A., Zdanavičienė E., Žilinskas J. (2015). *Candida albicans* importance to denture wearers. A literature review. Stomatologija.

[3] Delaloye J., Calandra T. (2014). Invasive candidiasis as a cause of sepsis in the critically ill patient. Virulence.

[4] Salerno C., Pascale M., Contaldo M., Esposito V., Busciolano M., Milillo L., Guida A., Petruzzi M., Serpico R. (2011). Candida-associated denture stomatitis. Med. Oral Patol. Oral Cir. Bucal.

[5] Gendreau L., Loewy Z.G. (2011). Epidemiology and etiology of denture stomatitis. J. Prosthodont..

[6] Barani K., Manipal S., Prabu D., Ahmed A., Adusumilli P., Jeevika C. (2014). Anti-fungal activity of *Morinda citrifolia* (noni) extracts against *Candida albicans*: An in vitro study. Indian J. Dent. Res..

[7] Gharechahi J., Asadzadeh N., Shahabian F., Gharechahi M. (2014). Flexural strength of acrylic resin denture bases processed by two different methods. J. Dent. Res. Dent. Clin. Dent. Prospect..

[8] Pattanaik S., BVJ V., Pattanaik B., Sahu S., Lodam S. (2010). Denture stomatitis: A literature review. J. Indian Acad. Oral Med. Radiol..

[9] Pellizzaro D., Polyzois G., Machado A.L., Giampaolo E.T., Sanitá P.V., Vergani C.E. (2012). Effectiveness of mechanical brushing with different denture cleansing agents in reducing in vitro *Candida albicans* biofilm viability. Braz. Dent. J..

[10] Lamfon H., Porter S.R., McCullough M., Pratten J. (2004). Susceptibility of *Candida albicans* biofilms grown in a constant depth film fermentor to chlorhexidine, fluconazole and miconazole: A longitudinal study. J. Antimicrob. Chemother..

[11] Depaola L.G., Spolarich A.E. (2007). Safety and efficacy of antimicrobial mouthrinses in clinical practice. J. Dent. Hyg..

[12] Zanatta F.B., Antoniazzi R.P., Rosing C.K. (2010). Staining and calculus formation after 0.12% chlorhexidine rinses in plaque free and plaque covered surfaces: A randomized trial. J. Appl. Oral Sci..

[13] Samaranayake L.P., McCourtie J., MacFarlane T.W. (1980). Factors affecting the in-vitro adherence of *Candida albicans* to acrylic surfaces. Arch. Oral Biol..

[14] Puttipan R., Wanachantararak P., Khongkhunthian S., Okonogi S. (2017). Effects of *Caesalpinia sappan* on pathogenic bacteria causing dental caries and gingivitis. Drug Discov. Ther..

[15] Villinski J.R., Bergeron C., Cannistra J.C., Gloer J.B., Coleman C.M., Ferreira D., Azelmat J., Grenier D., Gafner S. (2014). Pyrano-isoflavans from *Glycyrrhiza uralensis* with antibacterial activity against *Streptococcus mutans* and *Porphyromonas gingivalis*. J. Nat. Prod..

[16] Oliveira S.A.C., Zambrana J.R.M., Di Iorio F.B.R., Pereira C.A., Jorge A.O.C. (2014). The antimicrobial effects of *Citrus limonum* and *Citrus aurantium* essential oils on multi-species biofilms. Braz. Oral Res..

[17] Phumat P., Khongkhunthian S., Wanachantararak P., Okonogi S. (2017). Potential of *Piper betle* extracts on inhibition of oral pathogens. Drug Discov. Ther..

[18] Shoji Y., Nakashima H. (2004). Nutraceutics and delivery systems. J. Drug Target..

[19] Savjani K.T., Gajjar A.K., Savjani J.K. (2012). Drug solubility: Importance and enhancement techniques. ISRN Pharm..

[20] Kulthe S.S., Choudhari Y.M., Inamdar N.N., Mourya V. (2012). Polymeric micelles: Authoritative aspects for drug delivery. Des. Monomers Polym..

[21] Cagel M., Tesan F.C., Bernabeu E., Salgueiro M.J., Zubillaga M.B., Moretton M.A., Chiappetta D.A. (2017). Polymeric mixed micelles as nanomedicines: Achievements and perspectives. Eur. J. Pharm. Biopharm..

[22] Sousa F., Ferreira D., Reis S., Costa P. (2020). Current insights on antifungal therapy: Novel nanotechnology approaches for drug delivery systems and new drugs from natural sources. Pharmaceuticals.

[23] Oerlemans C., Bult W., Bos M., Storm G., Nijsen J.F.W., Hennink W.E. (2010). Polymeric micelles in anticancer therapy: Targeting, imaging and triggered release. Pharm. Res..

[24] Khonkarn R., Mankhetkorn S., Talelli M., Hennink W.E., Okonogi S. (2012). Cytostatic effect of xanthone-loaded mPEG-b-p(HPMAm-Lac 2) micelles towards doxorubicin sensitive and resistant cancer cells. Colloids Surf. B Biointerfaces.

[25] Bodratti A.M., Alexandridis P. (2018). Formulation of poloxamers for drug delivery. J. Funct. Biomater..

[26] Tima S., Anuchapreeda S., Ampasavate C., Berkland C., Okonogi S. (2017). Stable curcumin-loaded polymeric micellar formulation for enhancing cellular uptake and cytotoxicity to FLT3 overexpressing EoL-1 leukemic cells. Eur. J. Pharm. Biopharm..

[27] Anantaworasakul P., Okonogi S. (2017). Encapsulation of *Sesbania grandiflora* extract in polymeric micelles to enhance its solubility, stability, and antibacterial activity. J. Microencapsul..

[28] Caesar L.K., Cech N.B. (2019). Synergy and antagonism in natural product extracts: When 1 + 1 does not equal 2. Nat. Prod. Rep..

[29] Hussein Y.H.A., Youssry M. (2018). Polymeric micelles of biodegradable diblock copolymers: Enhanced encapsulation of hydrophobic drugs. Materials.

[30] Sezgin Z., Yüksel N., Baykara T. (2006). Preparation and characterization of polymeric micelles for solubilization of poorly soluble anticancer drugs. Eur. J. Pharm. Biopharm..

[31] Phumat P., Khongkhunthian S., Wanachantararak P., Okonogi S. (2018). Effects of *Piper betle* fractionated extracts on inhibition of *Streptococcus mutans* and *Streptococcus intermedius*. Drug Discov. Ther..

[32] Kitchener B.G.B., Wainwright J., Parsons A.J. (2017). A review of the principles of turbidity measurement. Prog. Phys. Geogr..

[33] Anderson C.W., Anderson C.W. (2005). Turbidity. Techniques of Water-Resources Investigations.

[34] Liu J., Xiao Y., Allen C. (2004). Polymer-drug compatibility: A guide to the development of delivery systems for the anticancer agent, ellipticine. J. Pharm. Sci..

[35] Mahmood S., Taher M., Mandal U.K. (2014). uma. Experimental design and optimization of raloxifene hydrochloride loaded nanotransfersomes for transdermal application. Int. J. Nanomed..

[36] Bhattacharjee S. (2016). DLS and zeta potential-What they are and what they are not?. J. Control. Release.

[37] Honary S., Zahir F. (2013). Effect of zeta potential on the properties of nano-drug delivery systems-A review (Part 1). Trop. J. Pharm. Res..

[38] Honary S., Zahir F. (2013). Effect of zeta potential on the properties of nano-drug delivery systems-A review (Part 2). Trop. J. Pharm. Res..

[39] Shaarani S., Hamid S.S., Kaus N.H.M. (2017). The Influence of pluronic F68 and F127 nanocarrier on physicochemical properties, in vitro release, and antiproliferative activity of thymoquinone drug. Pharmacogn. Res..

[40] Xie S., Tao Y., Pan Y., Qu W., Cheng G., Huang L., Chen D., Wang X., Liu Z., Yuan Z. (2014). Biodegradable nanoparticles for intracellular delivery of antimicrobial agents. J. Control. Release.

[41] Moreno-Rodríguez A.C., Torrado-Durán S., Molero G., García-Rodríguez J.J., Torrado-Santiago S. (2015). Efficacy and toxicity evaluation of new amphotericin B micelle systems for brain fungal infections. Int. J. Pharm..

[42] Keepers T.R., Gomez M., Celeri C., Nichols W.W., Krause K.M. (2014). Bactericidal activity, absence of serum effect, and time-kill kinetics of ceftazidime-avibactam against β-lactamase-producing Enterobacteriaceae and *Pseudomonas aeruginosa*. Antimicrob. Agents Chemother..

[43] Letscher-Bru V., Herbrecht R. (2003). Caspofungin: The first representative of a new antifungal class. J. Antimicrob. Chemother..

[44] Georgopapadakou N.H., Walsh T.J. (1996). Antifungal agents: Chemotherapeutic targets and immunologic strategies. Antimicrob. Agents Chemother..

[45] Kumar A., Zarychanski R., Pisipati A., Kumar A., Kethireddy S., Bow E.J. (2018). Fungicidal versus fungistatic therapy of invasive *Candida* infection in non-neutropenic adults: A meta-analysis. Mycology.

[46] Bradley S.G., Jones L.A. (1960). Mechnisms of action of antibiotics. Ann. N. Y. Acad. Sci..

[47] Hammond S.M. (1977). Biological activity of polyene antibiotics. Prog. Med. Chem..

[48] Sharma P., Jha A.B., Dubey R.S., Pessarakli M. (2012). Reactive oxygen species, oxidative damage, and antioxidative defense mechanism in plants under stressful conditions. J. Bot..

[49] Zhou L., Zhang P., Chen Z., Cai S., Jing T., Fan H., Mo F., Zhang J., Lin R. (2017). Preparation, characterization, and evaluation of amphotericin B-loaded MPEG-PCL-g-PEI micelles for local treatment of oral *Candida albicans*. Int. J. Nanomed..

[50] Daneshmand S., Golmohammadzadeh S., Jaafari M.R., Movaffagh J., Rezaee M., Sahebkar A., Malaekeh-Nikouei B. (2018). Encapsulation challenges, the substantial issue in solid lipid nanoparticles characterization. J. Cell. Biochem..

[51] Amini Y., Amel Jamehdar S., Sadri K., Zare S., Musavi D., Tafaghodi M. (2017). Different methods to determine the encapsulation efficiency of protein in PLGA nanoparticles. Biomed. Mater. Eng..

[52] Moffa E.B., Izumida F.E., Jorge J.H., Mussi M.C.M., Siqueira W.L., Giampaolo E.T. (2016). Effectiveness of chemical disinfection on biofilms of relined dentures: A randomized clinical trial. Am. J. Dent..

[53] Quirynen M., Bollen C.M.L. (1995). The influence of surface roughness and surface-free energy on supra- and subgingival plaque formation in man: A review of the literature. J. Clin. Periodontol..

[54] Radford D.R., Challacombe S.J., Walter J.D. (1999). Denture plaque and adherence of *Candida albicans* to denture-base materials in vivo and in vitro. Crit. Rev. Oral Biol. Med..

[55] Bollenl C.M.L., Lambrechts P., Quirynen M. (1997). Comparison of surface roughness of oral hard materials to the threshold surface roughness for bacterial plaque retention: A review of the literature. Dent. Mater..

[56] Da Silva F.C., Kimpara E.T., Mancini M.N.G., Balducci I., Jorge A.O.C., Koga-Ito C.Y. (2008). Effectiveness of six different disinfectants on removing five microbial species and effects on the topographic characteristics of acrylic resin. J. Prosthodont..

[57] Souza C.J.F., da Costa A.R., Souza C.F., Tosin F.F.S., Garcia-Rojas E.E. (2018). Complex coacervation between lysozyme and pectin: Effect of pH, salt, and biopolymer ratio. Int. J. Biol. Macromol..

[58] Phumat P., Khongkhunthian S., Wanachantararak P., Okonogi S. (2020). Comparative inhibitory effects of 4-allylpyrocatechol isolated from *Piper betle* on *Streptococcus intermedius*, *Streptococcus mutans*, and *Candida albicans*. Arch. Oral Biol..

[59] Naksuriya O., Shi Y., Van Nostrum C.F., Anuchapreeda S., Hennink W.E., Okonogi S. (2015). HPMA-based polymeric micelles for curcumin solubilization and inhibition of cancer cell growth. Eur. J. Pharm. Biopharm..

[60] Swaney A.C., Paffenbarger G.C., Caul H.J., Sweeney W.T. (1953). American dental association specification No. 12 for denture base resin: Second revision. J. Am. Dent. Assoc..

